# Influence of African Swine Fever Virus on Host Gene Transcription within Peripheral Blood Mononuclear Cells from Infected Pigs

**DOI:** 10.3390/v14102147

**Published:** 2022-09-29

**Authors:** Ann Sofie Olesen, Miyako Kodama, Kerstin Skovgaard, Ask Møbjerg, Louise Lohse, Morten T. Limborg, Anette Bøtner, Graham J. Belsham

**Affiliations:** 1 Section of Veterinary Clinical Microbiology, Department of Veterinary and Animal Sciences, University of Copenhagen, 1870 Frederiksberg, Denmark; 2 Department of Virus & Microbiological Special Diagnostics, Statens Serum Institut, 2300 Copenhagen, Denmark; 3 Center for Evolutionary Hologenomics, The Globe Institute, University of Copenhagen, 1350 Copenhagen, Denmark; 4 Department of Biotechnology and Biomedicine, Technical University of Denmark, 2800 Kongens Lyngby, Denmark

**Keywords:** host response, mRNA, transcription, host defence

## Abstract

African swine fever virus (ASFV) has become a global threat to the pig production industry and has caused enormous economic losses in many countries in recent years. Peripheral blood mononuclear cells (PBMCs) from pigs infected with ASFV not only express ASFV genes (almost 200 in number) but have altered patterns of host gene expression as well. Both up- and down-regulation of host cell gene expression can be followed using RNAseq on poly(A)+ mRNAs harvested from the PBMCs of pigs collected at different times post-infection. Consistent with the time course of changes in viral gene expression, only few and limited changes in host gene expression were detected at 3 days post-infection (dpi), but by 6 dpi, marked changes in the expression of over 1300 host genes were apparent. This was co-incident with the major increase in viral gene expression. The majority of the changes in host gene expression were up-regulation, but many down-regulated genes were also identified. The patterns of changes in gene expression within the PBMCs detected by RNAseq were similar in each of the four infected pigs. Furthermore, changes in the expression of about twenty selected host genes, known to be important in host defence and inflammatory responses, were confirmed using high-throughput microfluidic qPCR assays.

## 1. Introduction

African swine fever virus (ASFV) is the sole member of the *Asfarviridae* family and has a large, linear, dsDNA genome (ca. 170–190 kbp, depending on the strain) that includes nearly 200 genes (reviewed in [1]). This virus infects and causes disease in domestic pigs and in wild boar [2] but can also infect, apparently asymptomatically, other African wildlife species (family *Suidae*), including bush pigs and warthogs. In addition, ASFV can replicate within soft ticks (genus *Ornithodoros*) and is the only known DNA arbovirus. A sylvatic cycle involving replication in soft ticks and warthogs occurs in Africa [2]. Outside of Africa, transmission of the virus occurs mainly by direct or indirect contact between infected pigs or wild boar, generally without the involvement of soft ticks; however, some aspects of virus transmission are not well understood [3].

At least 24 different genotypes of the virus exist in Africa, and these are differentiated based on the sequence of the VP72 gene [4,5,6,7,8]. In 1957 and 1960, a genotype I ASFV spread from Africa into Europe (Portugal) and the virus remained present in the Iberian peninsula until the 1990s [9]. In 2007, a genotype II virus entered Georgia (in the Caucasus region), and subsequently, ASFV became widespread within neighbouring countries, i.e., in Russia and Eastern Europe. It also spread into Western Europe, including Belgium in 2018 (but now declared free again since 2020), and then into Germany in 2020 and Italy in 2021 [10]. Furthermore, in 2018, essentially the same virus was reported in China, the world’s largest pig producer [10,11], where it has had a major effect on pig production. The virus then quickly moved into many countries in the vicinity (e.g., Vietnam, Korea and Cambodia) and to the Philippines. In 2021, the virus was introduced into pigs in the Dominican Republic and Haiti [10], and thus, this virus is a major global concern.

Infection with ASFV can cause very high (near 100%) levels of case fatality in domestic pigs, and thus it has major economic importance. There are no commercially available approved vaccines or antiviral agents to combat the disease and hence, control measures have been solely reliant on culling of infected animals, restrictions on animal movement and high biosecurity [12,13]. However, recently, a commercial, live attenuated vaccine to help control the disease was approved for restricted use in Vietnam [14].

In the pig, the initial sites of virus replication during a natural infection are the pharyngeal tonsils, while secondary sites include the spleen, lymph nodes and liver [1]. More specifically, the virus replicates mainly in cells of the monocyte macrophage lineage [15], including within the population of peripheral blood mononuclear cells (PBMCs).

ASFV replicates within the cytoplasm of infected cells and the virus encodes its own RNA polymerase and transcription factors. Like host cell mRNAs, the viral mRNA transcripts are capped at their 5′-termini and are post-transcriptionally modified at their 3′-termini to include a poly(A) tail [1]. Thus, poly(A)+ mRNA from ASFV-infected cells includes both host and viral mRNAs. An initial analysis of ASFV gene expression, using poly(A)+ RNA extracted from PBMCs of pigs infected with a genotype II ASFV from Poland, termed ASFV/POL/2015/Podlaskie, was described recently [16]. It was apparent that there was little virus gene expression in PBMCs from infected pigs at 3 days post-inoculation (dpi); however, by 6 dpi, high levels of transcription of viral genes and virus replication had occurred. We have now undertaken the analysis of the host gene expression within the same RNA samples purified from PBMCs of four acutely infected pigs throughout the time course of the infection.

## 2. Materials and Methods

### 2.1. RNA Purification from PBMCs and Gene Expression Analysis

As described previously [16], total RNA was extracted from the PBMCs of 4 male pigs (Landrace × Large White) that were inoculated intranasally with the genotype II ASFV, designated POL/2015/Podlaskie (GenBank accession number MH681419). Briefly, EDTA-stabilised blood (EDTA blood) samples were collected from each pig prior to inoculation on 0 dpi, and at 3 and 6 dpi. PBMCs were purified from the EDTA blood samples (4 mL) using the Histopaque^®^ system (Sigma-Aldrich, St. Louis, MO, USA) and lysed by the addition of Trizol^TM^ Reagent (Thermo Fisher Scientific, Waltham, MA, USA). Total RNA was extracted from the samples in the Trizol^TM^ Reagent using the Direct-zol^TM^ RNA MiniPrep kit (Zymo Research, Irvine, CA, USA). This system includes a DNAse I digestion to remove host and viral DNA. Analysis of the RNA transcripts by RNAseq was performed using poly(A)+ selected mRNAs (these samples include both viral and host mRNAs, but the selection removes most ribosomal RNA) that were then sequenced (following reverse transcription using random primers, second strand synthesis, adaptor ligation and PCR amplification) by the BGI Europe Genome Centre (Copenhagen, Denmark) (termed RNA-T on DNBseq, aiming at about 40 million reads per sample).

### 2.2. Mapping of Sequence Reads to the Pig Genome

Sequence reads (27–47 million per sample) were mapped using STAR v. 2.7.0 [17] to the USMARCv1.0 pig genome assembly (accession no. PRJNA392765), which was derived from a male pig within a population that was approximately one-half Landrace, one-quarter Duroc and one-quarter Yorkshire (see [18]). The counts were then normalised, when indicated, to take account of the library size and the length of each gene from each sample (gene length corrected trimmed mean of M-values (GeTMM)) [19].

### 2.3. Differential Gene Expression Analyses

Differential gene expression analyses were performed on raw read counts using several Bioconductor packages, namely DESeq2 [20], EdgeR using glmQL models [21,22] and Limma [23]. A threshold of ≤0.05 was set for the adjusted *p*-value or false discovery rate used to determine whether a gene was differentially expressed.

MA plots, PCA plots and Venn diagrams were made in R and figures were assembled in CorelDraw (Corel Corporation, Austin, TX, USA).

### 2.4. High-Throughput RT-qPCR

The total RNA samples from the PBMCs were also used for analysis of selected genes using an array of reverse transcription (RT) quantitative real-time PCRs (qPCRs), essentially as described previously [24]. In brief, total RNA (200 ng) was converted into cDNA by reverse transcription using the QuantiTect Reverse Transcription Kit (Qiagen, Hilden, Germany). The cDNA was diluted 1:10 in low-EDTA TE buffer prior to 19 cycles of pre-amplification, followed by exonuclease treatment as described previously [25]. Pre-amplified cDNA was diluted 1:10 in low-EDTA TE buffer prior to the qPCRs. The panel of genes for analysis was chosen based on the RNAseq results plus previous results with samples from influenza virus-infected pigs [24]. For the new assays, primers were designed using Primer3 (http://bioinfo.ut.ee/primer3–0.4.0/) and purchased from Sigma-Aldrich. Primer sequences are shown in Supplementary Table S1. The qPCRs were carried out in Dynamic Array Integrated Fluidic Circuit chips on the BioMark HD real-time PCR platform (Fluidigm), as described previously [25]. Cq values were acquired using the Fluidigm Real-Time PCR Analysis software 3.0.2 (Fluidigm) and exported to GenEx6 (MultiD) for data processing, including correction for PCR efficiency, normalisation to reference genes and averaging of cDNA technical repeats. Using geNorm and NormFinder algorithms (in GenEx), beta-2-microglobulin (B2M), peptidylprolyl isomerase A (PPIA), HPRT1 and YWHAE were selected as reference genes and used for data normalisation.

## 3. Results

In results described previously [16], the four pigs (numbered 9–12) that were inoculated at 0 dpi with ASFV POL/2015/Podlaskie all displayed fever from 4 or 5 days post-inoculation, and three of the four (except for pig 9) displayed clear clinical signs of infection at 6 dpi. Blood samples were collected from each of the four pigs prior to inoculation on 0 dpi and also at 3 and 6 dpi. From these 12 blood samples, PBMCs were isolated and RNA was extracted. The poly(A)+ mRNAs were purified from the total RNA preparations and sequenced (see Materials and Methods). Approximately 81–92% of the sequence reads mapped uniquely to the pig genome [16]. The analysis of these host-derived reads derived from PBMCs throughout the course of the infection in the pigs is presented here. The expression of ASFV genes in the PBMCs from these inoculated animals was reported previously [16].

### 3.1. Principal Component Analysis (PCA)

**Figure 1:**
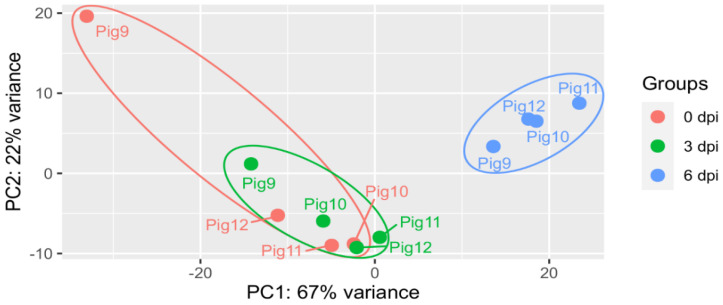
PCA across different sampling days. Pigs were inoculated intranasally with ASFV/POL/2015/Podlaskie at 0 dpi and euthanised at 6 dpi. Blood samples were collected at 0 dpi (prior to inoculation), at 3 dpi and at 6 dpi (before euthanasia) from each of the 4 pigs. PBMCs were purified from the blood and poly(A)+ mRNA purified and analysed by sequencing (see Materials and Methods). Approximately 27–42 million reads were obtained from each sample and assessed using PCA. The samples from each pig (9–12) on each sampling day are indicated.

As an initial assessment of the host gene sequence reads generated by RNAseq, PCA was performed on the data from across the different sampling days (Figure 1). In general, on each day, the data from the four pigs grouped closely together (the one exception was pig 9 on day 0, which was more distinct, perhaps because it had one of the smaller numbers of uniquely mapped reads and did not meet the recommended quality standards of BGI). There was a large overlap between the groups identified at day 0 and 3 dpi. However, strikingly, the reads from the PBMCs at 6 dpi formed a distinct cluster from the other samples, indicating a markedly different population of mRNAs present within these samples.

### 3.2. Differential Gene Expression Analysis

**Figure 2:**
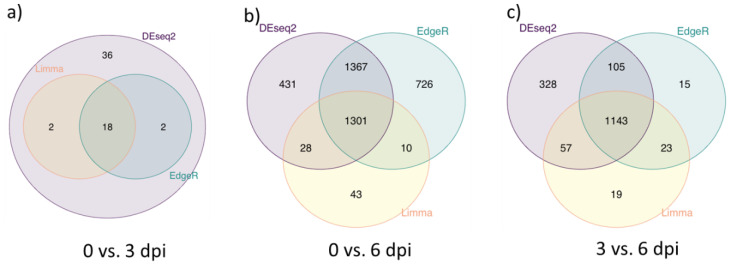
Differential gene expression analyses across 3 pairwise comparisons using 3 different methods. Venn diagrams of the differentially expressed genes (DEGs) in PBMCs from pigs collected at 0 dpi, 3 dpi and 6 dpi. (**a**) Venn diagram showing the overlap between the DEGs identified by the DEseq2, glmQL for EdgeR and Limma packages (see Materials and Methods) shown in lilac, blue and yellow, respectively, between PBMCs from the uninfected (0 dpi) and infected pigs at 3 dpi. (**b**) Venn diagram of the DEGs detected by these same packages between the PBMCs from uninfected pigs (0 dpi) and from infected pigs at 6 dpi. (**c**) Venn diagram of the DEGs between PBMCs collected from pigs at 3 and 6 dpi.

Three different software tools were used to compare the differential expression (DE) of mRNAs at the different stages of infection. The overall consistency in identification of differentially expressed genes (DEGs) by the Bioconductor tools was high and is shown in Figure 2, but each tool identified a slightly different population of changes in gene expression. Only genes showing a greater than 2-fold difference in gene counts were scored as DEGs in this analysis. It was apparent that there were few DEGs in the PBMCs at 3 dpi compared to the PBMCs from the pigs at 0 dpi (Figure 2a). Just 18 genes were identified, in common, by each of the three Bioconductor tools. However, in contrast, over 1300 genes were identified as being differentially expressed in the PBMCs of pigs collected at 6 dpi compared to the 0 dpi samples (Figure 2b). Consistent with these results, over 1100 genes were scored as differentially expressed between 3 and 6 dpi (Figure 2c).

### 3.3. Changes in Host Gene Expression in PBMCs from ASFV-Infected Pigs

**Figure 3:**
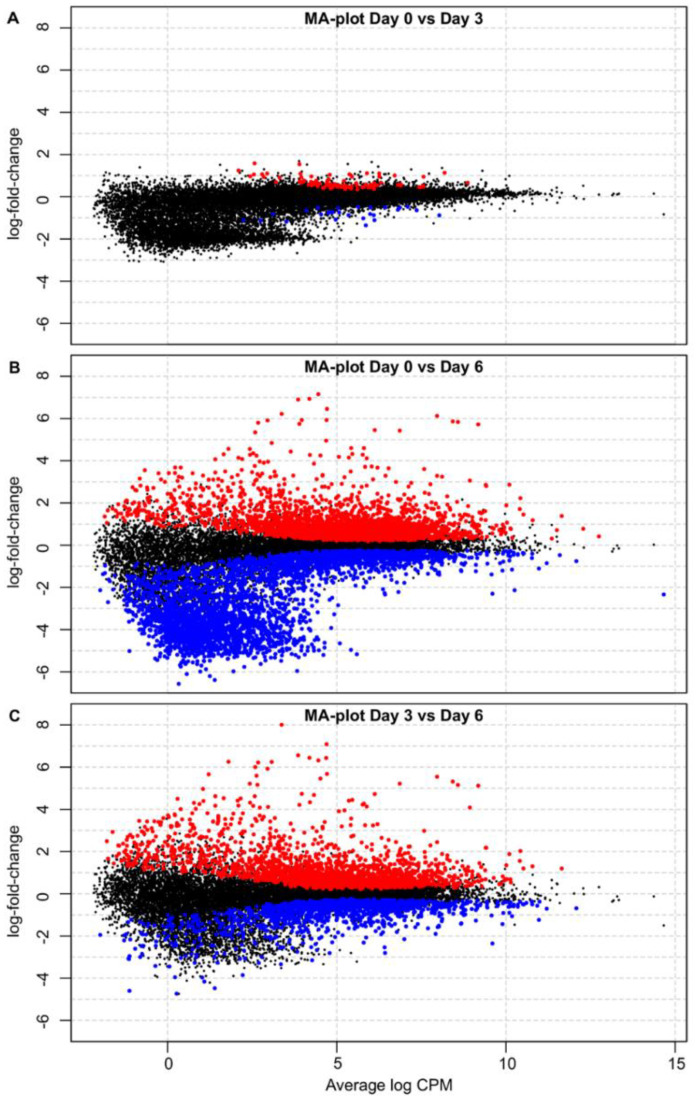
Changes in host gene expression in PBMCs from ASFV-infected pigs. MA plots showing the log_2_-fold change in mean gene expression (average log_2_ counts per million (CPM) at different times post-inoculation. (**A**) PBMCs from pigs 0 dpi vs. 3 dpi. (**B**) PBMCs from pigs at 0 dpi vs. 6 dpi. (**C**) PBMCs from pigs at 3 dpi vs. 6 dpi. Red and blue data points show genes that were found by EdgeR to be expressed at a significantly higher or lower level, respectively. Black data points show genes that are not significantly changed. The plots were made using the glmQL model in EdgeR and assembled in CorelDRAW.

The host gene expression was compared, using EdgeR, in PBMCs taken from the pigs at 0, 3 and 6 dpi and displayed using MA plots (Figure 3). At 3 dpi, few changes in gene expression were apparent (Figure 3A). Both up-regulation (shown in red) and down-regulation (in blue) were observed, but these changes were generally small in magnitude (only about 1 log_2_ change). In contrast, at 6 dpi, a large number of genes were differentially expressed (Figure 3B). As may be expected from the great change in the level of virus replication that occurred in this period [16], most of the significant changes in gene expression occurred between 3 and 6 dpi (Figure 3C). Many (>1100) different host genes were markedly changed in their expression during this time (Figure 2c), either increased or decreased.

### 3.4. Analysis of the Expression of Individual Genes within PBMCs throughout the Course of ASFV Infection in Pigs

The changes in gene expression were determined from the normalised numbers of sequence reads using three different Bioconductor tools. About 75% of the changes in gene expression were increases, and the remainder were reductions. Lists of the 20 genes showing the most pronounced decreases and increases in expression, determined by the three different Bioconductor tools, are shown in the Supplementary Information (Tables S2 and S3, respectively). Examples of genes showing increased expression include CXCL8 (interleukin (IL)-8), CCL2, GPR84 and S100A8. Plots showing the marked increase in expression of some selected individual genes throughout the course of the infection in the four separate pigs are shown in Figure S1a–g. In contrast, a plot showing the decreased expression of the PCD1B gene, encoding the CD1B antigen, and the NOX3 gene at 6 dpi in all four pigs is shown in Figure S1h,i. It is apparent that certain genes, e.g., FAM111B and CXHXorf21 and CHMP7 (see Figure S1a–c) were partially or fully enhanced in their expression at 3 dpi, whereas others showed little change until 6 dpi (e.g., CXCL8 and S100A8, both involved in inflammatory responses; see Figure S1f,g). Strikingly, the pattern of changes in gene expression for each of the genes shown was similar in each of the four pigs (Figure S1a–i).

### 3.5. Changes in Selected Host Gene Expression in PBMCs from Pigs That Were Infected with ASFV

**Figure 4:**
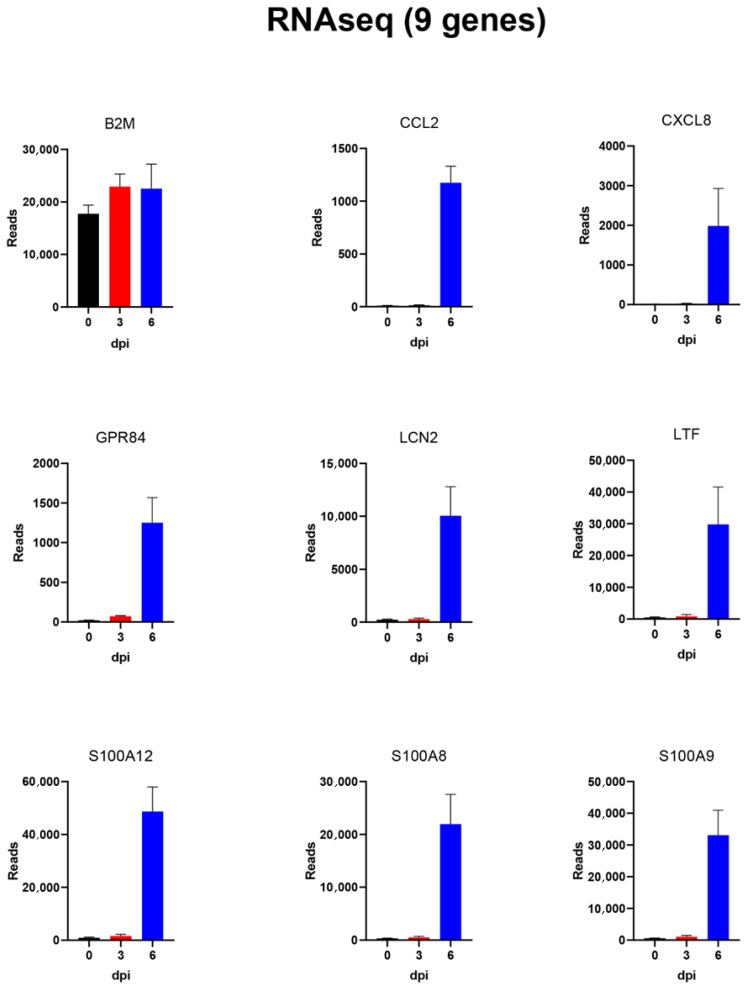
Expression of 9 selected host genes in PBMCs from ASFV-infected pigs as determined by RNAseq. The normalised numbers of gene sequence reads (mean + SEM for 4 pigs) for these selected genes are shown for the PBMCs collected at 0, 3 and 6 dpi. The expression of B2M was expected to be unchanged and acted as a reference gene. All of the other genes were expressed at >50–100-fold higher levels at 6 dpi compared to 0 dpi. There was little or no change in expression of these genes at 3 dpi.

**Figure 5:**
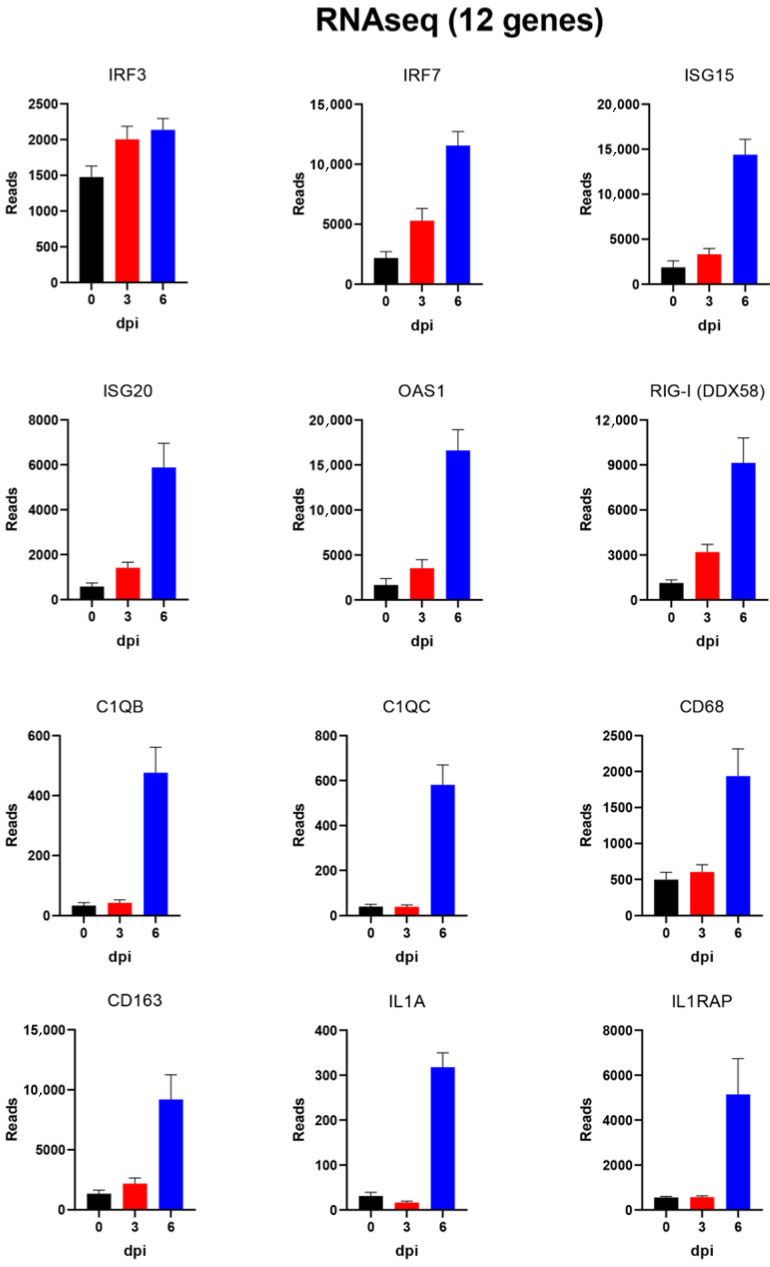
Expression of 12 selected host genes in PBMCs from ASFV-infected pigs as determined by RNAseq. The normalised numbers of gene sequence reads (mean of 4 pigs +SEM) for these selected genes are shown for the PBMCs collected at 0, 3 and 6 dpi. All of these genes (except for IRF3) were expressed at >3–10-fold higher levels at 6 dpi compared to 0 dpi.

To confirm the results of the RNAseq analysis, the expression of a selection of almost 40 different host genes (including reference genes) was also analysed using microfluidic qPCRs (see below). For ease of comparison, the results for this selected set of genes, as determined by RNAseq, are shown in Table 1, while the results for the qPCR assays are shown in Table 2. Graphical representations of the changes in expression detected by RNAseq for some of these selected genes are shown in Figure 4 and Figure 5. The mean values (from the four pigs) of the normalised reads for eight genes, whose expression was greatly increased (see Table 1), along with B2M (beta-2 microglobulin) acting as a reference gene, are shown in Figure 4. The expression of the reference gene was barely changed throughout the course of the infection, as expected. In contrast, the expression of CCL2, CXCL8, GPR84, LCN2, LTF, S100A12, S100A8 and S100A9 was greatly enhanced (ca. 50–100-fold) at 6 dpi, but with little or no change observed at 3 dpi. Expression levels of a further 12 genes are shown in Figure 5; the changes in expression of these genes were more moderate than those shown in Figure 4. These included a number of genes that contribute to an antiviral state that are induced by type I and type III interferons (e.g., IRF7, ISG15, ISG20 and OAS) or more directly by the presence of dsRNA (i.e., RIG-I (DDX58)). There was little change in the expression of IRF3 during the course of the infection, but these other genes were expressed at ca. 5–10-fold elevated levels at 6 dpi (Figure 5) compared to 0 dpi. It is noteworthy that the expression levels of the interferon-sensitive genes IRF7, ISG15, ISG20 and OAS1 (and RIG-I, which is also involved in the interferon response) were slightly increased at 3 dpi, in each case, but the changes were much greater at 6 dpi (Figure 5 and Table 1).

### 3.6. Analysis of Changes in Gene Expression in PBMCs Using High-Throughput Microfluidic qPCR Assays

**Figure 6:**
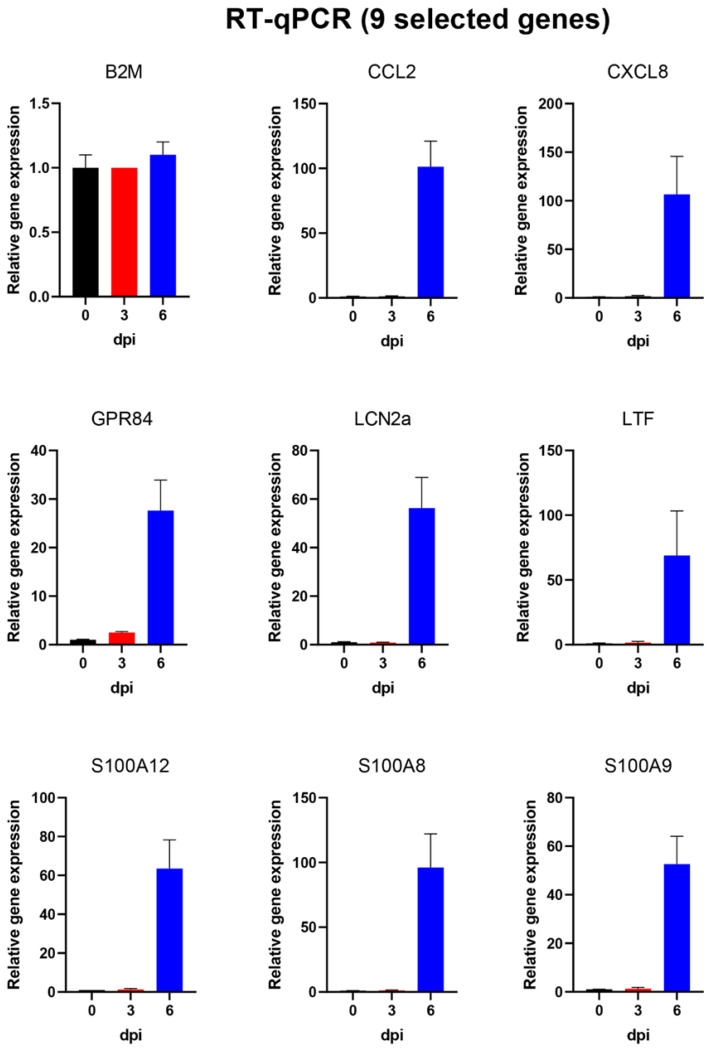
Expression of 9 selected host genes in PBMCs from ASFV-infected pigs as determined by RT-qPCR. The relative levels (mean + SEM for 4 pigs) of 9 selected genes, as determined by qPCR assays, are shown for the PBMCs collected at 0, 3 and 6 dpi. The expression of B2M was expected to be unchanged and acted as a reference gene. All the other genes shown here were expressed at about 30–100-fold higher levels at 6 dpi compared to 0 dpi.

**Figure 7:**
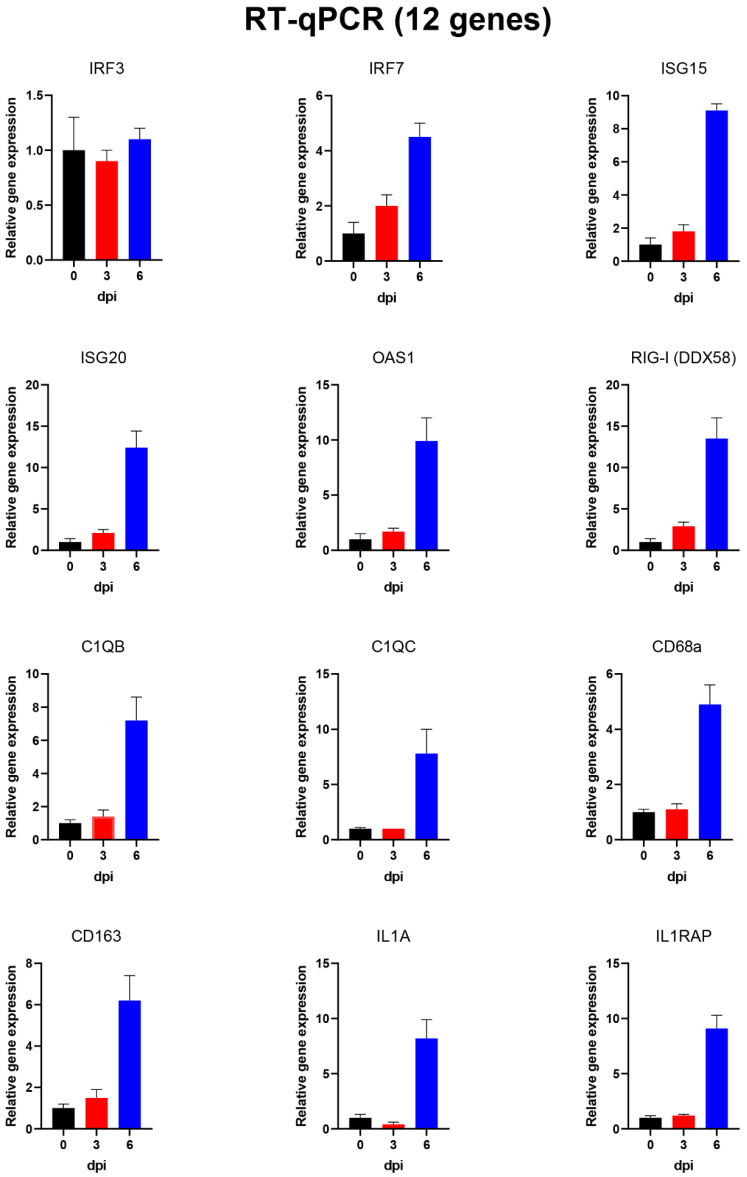
Expression of 12 selected host genes in PBMCs from ASFV-infected pigs. The relative expression levels (mean of 4 pigs +SEM) for these selected genes, as determined by qPCR assays, are shown for the PBMCs collected at 0, 3 and 6 dpi. All the genes (except for IRF3) were expressed at 4–12-fold higher levels at 6 dpi compared to 0 dpi.

As indicated above, the RNA samples prepared from the purified PBMCs were also assayed for expression of 37 different genes (including some reference genes selected for standardisation purposes) using high-throughput qPCR assays (see Table 2). The genes analysed in this way were chosen on the basis of the changes observed by RNAseq and the availability of validated assays (as used previously for studies on influenza virus-infected pigs [24]). The results of some of these analyses are shown in Figure 6 and Figure 7 for the same sets of genes shown in Figure 4 and Figure 5 from the RNAseq analyses. As expected, the B2M reference gene was again found to be expressed at similar levels throughout the infection period. In addition, consistent with the RNAseq results, expression of some of the selected genes was shown (see Figure 6) to be strongly increased (up to 100-fold) at 6 dpi (i.e., CCL2, CXCL8, GPR84, LCN2, LTF, S100A12, S100A8 and S100A9). Furthermore, expression levels of a further 11 genes, including various genes involved in the development of an antiviral state, were shown to be markedly enhanced (>3–10-fold) at 6 dpi, while IRF3 expression was not changed (Figure 7). It is apparent that the two approaches to analyse gene expression, using RNAseq (Figure 4 and Figure 5) and qPCR assays (Figure 6 and Figure 7), provided similar patterns of results.

None of the genes selected for analysis in the microfluidic qPCR system showed a clear decrease in expression during the experiment (Figure 6 and Figure 7, plus Table 2). The reference genes, e.g., B2M (see Figure 6) and PPIA, which were included in the qPCR analysis, remained fairly constant throughout the experiment (see Table 2), as expected.

## 4. Discussion

There are important differences between measuring the changes in virus gene expression within cells of an infected animal and determining changes in host gene expression in the same samples. Firstly, the number of viral genes is much smaller than the number of host genes—even for a large DNA virus such as ASFV, there are less than 200 genes that are expressed from the viral genome, while the pig genome includes over 20,000 protein-coding genes. Secondly, within uninfected cells, there is no expression of ASFV genes, whereas for the host genes, thousands of different mRNAs will be present initially within the cells, when infection occurs, then either increases or decreases in expression can occur.

Changes in host gene expression detected in the PBMCs can be a direct effect of the virus upon the host cells, but they can also reflect indirect changes caused by changes in intercellular signalling, e.g., in response to cytokines such as interferon. Changes in cell populations within the animal, e.g., loss or gain of particular cell types, can also affect, in principle, the overall expression level of specific genes within the populations of cells that make up the PBMCs.

In contrast to experiments performed in cell culture, it is not possible to precisely synchronise the infection of cells within a live animal, nor is it possible to ensure that all susceptible cells within the animal will become infected. Thus, it is interesting to note the high degree of correspondence in the changes observed among the four different pigs used in this study. As observed with the virus-encoded transcripts [16], there was high concordance between the changes in host gene expression observed in the four different pigs (e.g., see Figure S1); this was true both in terms of the time course and also the magnitude of the changes.

Some of the genes in the PBMCs that were most highly increased in expression at 6 dpi are well-known cytokine genes such as CCL2 and CXCL8. A variety of other genes known to have a role in inflammatory responses (e.g., IL1A and IL1RAP) and antiviral responses (ISG15, ISG20 and RIG-I) were also more highly expressed. These changes in gene expression were detected initially by the RNAseq analysis (Table 1, Figure 4 and Figure 5) but were then confirmed using microfluidic qPCRs (Table 2, Figure 6 and Figure 7). There is a high degree of agreement between the different types of assay for which genes are expressed at an elevated level or not. This increases confidence in both approaches, which each have their own advantages and disadvantages.

### 4.1. Key Changes in Host Gene Expression

The changes in gene expression measured here by qPCR indicate the extent of the change (as a relative change in gene expression). This has advantages from a comparative point of view, but it is not possible to predict whether a large change in gene expression will necessarily be significant. For example, a 100-fold change in the expression of a gene that is normally expressed at a low level may still only achieve a modest level of the mRNA, and this may or may not result in a significant change in the activity of the encoded protein within the cells. In contrast, a gene that is highly expressed in the cells of the uninfected pig may be significantly down-regulated, but the function of the encoded protein within the cell is largely preserved. The RNAseq approach provides a comprehensive data set (since it covers all viral and host genes and provides information about expression levels) but is more time-consuming.

### 4.2. Different Approaches to Assessing Changes in Host Gene Expression in Cells following ASFV Infection

Recently, Cackett et al. [26] described changes in the transcription of cellular genes in primary porcine alveolar macrophages that were infected in vitro with ASFV Georgia 2007/1 at an moi of 5 and analysed at 5 h (early) or 16 h (late) post-infection (hpi). The enhanced expression of several S100 family genes was observed as demonstrated here (Figure S1f, plus Table 1 and Table 2). However, they also reported that a number of important cytokines, including CXCL2 and CXCL8, were down-regulated between 5 and 16 hpi, which contrasts with the strong stimulation of expression (ca. 100-fold, see Figure 4) observed here for the CXCL8 gene observed within the PBMCs of the infected pigs at 6 dpi. No increases in the expression of ISG15 or TNF were observed in the lung macrophages either, while marked increases in ISG15 and other interferon response genes (ISG20 and OAS1) were observed in the PBMCs used here (in both RNAseq and qPCR assays), although there were only modest or no changes in TNF expression observed here (Table 1 and Table 2). The basis for these disparities in results are not clear, but it is apparent that there is a significant difference between infection of cells in culture, at high moi, and responses in circulating cells which may be exposed to a variety of external stimuli within an infected animal.

Our results overlap with, but also extend, the findings of Jaing et al. [27], who examined changes in gene expression within RNA from whole blood collected from three pigs infected with ASFV Georgia 2007/1 at 7–10 dpi. They also detected marked increases in the expression of S100A8 and S100A9 (as seen here, see Figure 4), and they observed increases in the expression of LTF (as also shown here, Figure 4). Since large increases in the expression of these genes occurred in the infected animals, it is not surprising that both studies detected these changes. However, it was noted previously [16] that in the study by Jaing et al. [27], among the three ASFV Georgia-infected pigs, there were variable levels of viral gene expression, making the interpretation of the changes they observed more difficult. This contrasted with the similar profiles of viral [16] and host (see Figure S1) gene expression observed among the four pigs studied here.

Fishbourne et al. [28] examined the expression of various cytokines and their receptors in whole blood from pigs infected with another highly virulent ASFV (Benin 97/1). They noted a large increase in the levels of CCL2 mRNA, as seen here. However, they also measured the levels of various cytokines in plasma but saw no increase in the level of CXCL8 protein in pigs infected with the virulent ASFV at 3 and 7 dpi compared to uninfected pigs. This is in contrast with the marked increase in expression of the mRNA from this gene in PBMCs observed here, but clearly, different things are being measured. No change in the level of interferon gamma (IFNG) was observed in the plasma of their animals either, and this is consistent with the lack of major change in the level of the corresponding mRNA observed here (Table 1 and Table 2).

The role of interferon in ASFV infections is not entirely clear. Low-virulence strains of the virus have been reported to induce the expression of interferon in macrophages in vitro, whereas virulent strains of ASFV do not [29,30]. However, type I interferons have been detected in serum of pigs infected with the virulent ASFV Georgia 2007/1 [31], which seems consistent with the elevated expression of various ISGs and IRFs seen here (Table 1 and Table 2). A more recent study by Golding et al. [32] confirmed the presence of interferon in pigs infected with ASFV Georgia 2007/1, coincident with the viremia. However, there was no apparent effect of interferon treatment on the replication of this strain in alveolar macrophages in vitro. Perhaps other cells are affected by the circulating interferons.

It was interesting to note that a number of genes involved in generating an anti-virus response, e.g., ISGs and IRFs, were seen to be elevated in their expression (albeit at a sub-maximal level) at 3 dpi, whereas genes involved in inflammatory responses (e.g., S100 family members) were only increased in expression at 6 dpi (see Figure 4, Figure 5, Figure 6 and Figure 7).

There are advantages and disadvantages of assessing changes in gene expression within cells that are infected in cell culture compared to harvesting cells from infected animals. Clearly, it is possible to define the nature and timing of the infection much more precisely in cell culture (as used by Cackett et al. [26,33]) rather than within live animals (as used here and by Olesen et al. [16] and others [27,28]). However, the latter is, of course, the situation that exists within the natural host animals. Primary cells in culture can change rapidly in their properties (including their gene expression), and the population of cells that are maintained in cell culture may not reflect the diversity of cells that is present initially within the animal. Furthermore, the effect of other changes in ASFV-infected animals (e.g., from fever reaching > 41 °C, or by signalling resulting from production of factors (e.g., cytokines) from other tissues) may not be replicated in vitro.

Some of the genes that were most strongly enhanced in their expression at 6 dpi were members of the S100 family of proteins. These are rather small (10–12 kDa) calcium-binding proteins that have a range of different functions related to inflammation [34]. The extracellular presence of these proteins is considered a possible biomarker for certain diseases, but it is not yet known whether the enhanced expression of these genes as mRNAs in PBMCs from ASFV-infected pigs results in elevated expression of the encoded proteins in blood—this depends on the secretion (or release) of these proteins from the cells. The analysis of changes in host gene expression described here provides multiple candidates for potential markers of active infection to complement detection of the virus itself.

## 5. Conclusions

The outcome of an infection by any agent (e.g., virus or bacteria, etc.) depends not only on the infecting organism itself but also on the host response to that infection, e.g., inflammation, fever, the acquired immune response and potentially the presence of other micro-organisms [35]. The host responses will generally lead to recovery from the infection but can also contribute to the disease. Thus, detailed knowledge of gene expression from the infectious agent in the host (as we described previously for ASFV [16]) and of the responses to the virus infection in the host (as described here) is required to understand the nature of the disease and to develop novel methods to combat it.

## Figures and Tables

**Table 1:** The mean values of normalised reads (plus SEM) for selected genes in the 4 pigs inoculated with ASFV in PBMCs collected at 0, 3 and 6 dpi as determined by RNAseq. Genes that were increased in expression at 6 dpi by >3-fold or >10-fold compared to 0 dpi are highlighted in yellow and green, respectively. Genes increased by ≥2-fold at 3 dpi are highlighted in blue; note that these genes are each involved in the generation of an anti-virus response.

Gene ^1^	Mean Reads 0 dpi	SEM	Mean Reads 3 dpi	SEM	Mean Reads 6 dpi	SEM
B2M	17,770	1617	22,921	2379	22,529	4663
C1QB	34	10	43	10	476	85
C1QBP	1554	194	2175	216	3049	249
C1QC	40	10	39	9	581	89
CCL2	11	3	15	3	1174	158
CCL4	413	37	417	43	1269	65
CD101	204	22	218	22	478	120
CD14	1462	234	2491	535	4529	989
CD163	1348	282	2176	482	9191	2069
CD68	497	101	603	103	1936	382
CXCL8	13	1	26	4	1984	946
CXCL9	9	4	10	2	87	23
CXCR2	340	106	501	177	4429	1481
RIG-I (DDX58)	1139	204	3195	507	9131	1674
GPR84	23	2	73	9	1251	317
IFNG	58	8	87	11	202	33
IL1A	31	8	17	3	318	32
IL1RAP	559	44	571	59	5142	1596
IRF3	1476	155	2004	183	2137	157
IRF7	2176	549	5297	1006	11,558	1163
ISG15	1868	748	3332	632	14,387	1723
ISG20	580	155	1417	240	5877	1077
LCN2	233	56	297	79	10,041	2749
LTF	516	182	847	601	29,764	11,781
MX1	9184	2963	21,212	3847	68,391	7402
MX2	1845	604	2820	554	8547	1365
OAS1	1681	693	3532	950	16,605	2317
OAS2	6640	1780	11,464	1791	24,732	4024
OASL	90	11	114	17	659	111
PPIA	26,158	3235	33,908	3636	43,087	3728
RPL13A	46,135	4072	55,115	4428	34,299	1795
S100A12	946	213	1612	605	48,702	9254
S100A8	311	80	524	190	21,969	5629
S100A9	577	116	1039	380	33,094	7872
TNF	109	11	184	21	453	86
VWF	1083	272	1332	426	3722	1223

^1^ A description of the function of each gene product may be obtained from the GeneCards website (https://www.genecards.org).

**Table 2:** The mean relative expression (RE) levels (plus SEM) of selected genes in the 4 pigs inoculated with ASFV in PBMCs collected at 0, 3 and 6 dpi as determined by qPCR assays. In each case, the relative expression of each gene in the pigs at 0 dpi was set to 1 and other results for each gene for that pig compared to this value. As in Table 1, genes that were increased in expression at 6 dpi by >3-fold or >10-fold compared to 0 dpi are highlighted in yellow and green, respectively. Genes increased by ≥2-fold at 3 dpi are highlighted in blue.

Gene ^1^	Mean RE 0 dpi	SEM	Mean RE 3 dpi	SEM	Mean RE 6 dpi	SEM
B2M	1.0	0.1	1.0	0	1.1	0.1
C1QB	1.0	0.2	1.4	0.4	7.2	1.4
C1QBP	1.0	0.1	1.1	0	1.3	0.1
C1QC	1.0	0.1	1.0	0	7.8	2.2
CCL2	1.0	0.3	1.2	0.4	101.3	19.8
CCL4	1.0	0.4	0.6	0.1	1.5	0.1
CD101	1.0	0.2	1.3	0.1	1.3	0.2
CD14	1.0	0.1	1.4	0.3	2.4	0.3
CD163	1.0	0.2	1.5	0.4	6.2	1.2
CD68a *	1.0	0.1	1.1	0.2	4.9	0.7
CD68b *	1.0	0.2	1.2	0.2	5.2	0.8
CXCL8	1.0	0.2	1.7	0.8	106.6	39.1
CXCL9	1.0	0.2	0.6	0.2	10.8	2.5
CXCR2	1.0	0.3	1.0	0.3	8.5	1.9
RIG-I (DDX58)	1.0	0.4	2.9	0.5	13.5	2.5
GPR84	1.0	0.1	2.5	0.2	27.6	6.3
IFNG	1.0	0.2	1.2	0.1	2.9	0.3
IL1A	1.0	0.3	0.4	0.2	8.2	1.7
IL1RAP	1.0	0.2	1.2	0.1	9.1	1.2
IRF3	1.0	0.3	0.9	0.1	1.1	0.1
IRF7	1.0	0.4	2.0	0.4	4.5	0.5
ISG15	1.0	0.4	1.8	0.4	9.1	1.3
ISG20	1.0	0.4	2.1	0.4	12.4	2.0
LCN2a *	1.0	0.2	0.8	0.2	43.2	8.1
LCN2b *	1.0	0.2	0.8	0.2	56.3	12.6
LTF	1.0	0.3	1.5	1.1	68.9	34.4
MX1	1.0	0.5	1.8	0.2	4.9	0.7
MX2	1.0	0.4	1.5	0.2	2.1	0.7
OAS1	1.0	0.5	1.7	0.3	9.9	2.1
OAS2	1.0	0.4	1.8	0.1	2.2	0.5
OASL	1.0	0.2	1.3	0.4	2.7	0.3
PPIA	1.0	0.1	1.0	0.1	1.3	0.1
RPL13A	1.0	0	0.9	0.1	0.4	0.1
S100A12	1.0	0	1.3	0.5	63.5	14.8
S100A8	1.0	0.1	1.2	0.4	96.0	26.0
S100A9	1.0	0.1	1.3	0.5	52.6	11.5
TNF	1.0	0.2	1.3	0.2	1.2	0.2
VWF	1.0	0.4	0.9	0.3	1.7	0.6

* Two separate qPCR assays were used to determine the expression of CD68 and LCN2, labelled as (a) and (b), respectively, and gave similar results in each case. ^1^ A description of the function of each gene product may be obtained from the GeneCards website (https://www.genecards.org).

## Data Availability

The data analysed in this study will be made available in a relevant open access repository upon acceptance.

## Supplementary Materials

The following supporting information can be downloaded at: https://www.mdpi.com/article/10.3390/v14102147/s1, Figure S1: Changes in gene expression in individual pigs. Table S1: List of primers used for the qPCRs to determine the levels of the cDNAs corresponding to the mRNAs transcribed from the indicated genes. Table S2: List of the 20 genes with the greatest reduction in expression within PBMCs, as determined by RNAseq, between pigs at 0 and 6 dpi. Table S3: List of the 20 genes with the most increased expression in PBMCs, as determined by RNAseq, between pigs at 0 and 6 dpi.
