# Supplementary material for: Influence of African Swine Fever Virus on Host Gene Transcription within Peripheral Blood Mononuclear Cells from Infected Pigs
Source: Viruses. 2022 Sep 29;14(10):2147. doi: 10.3390/v14102147 (PMC9610944; doi:10.3390/v14102147)
Supplement: Supplementary file 1 [file viruses-14-02147-s001.zip › viruses-1923885-Table S1.pdf]

viruses

MDPI

Table S1. List of primers used for the qPCRs to determine the levels of the cDNAs corresponding to the mRNAs transcribed from the indicated genes. Note two different assays were used for CD68 and LCN2 and are labelled (a) or (b) respectively.

Gene Symbol	Gene Name	Primer 1 Sequence (5' to 3')	Primer 2 Sequence (5' to 3')	Amplicon Length (bp)
B2M	Beta-2-Microglobulin	TGAAGCACGTGACTCTCGAT	CTCTGTGATGCCGGTTAGTG	70
C1QB	Complement C1q B Chain	GGGGATCAAGGGAGAGAAAG	CTCCCTTCTCTCCAACCTCA	68
C1QBP	Complement C1q Binding Protein	GCTGGAAGTGAATGGGACAG	GTGGGATGCTGTTGTTGATG	92
C1QC	Complement C1q C Chain	AGGATGGGCATGATGGACT	ATCTCCCTTCTCACCCTTGG	92
CCL2	C-C Motif Chemokine Ligand 2	CTTCTGCACCCAGGTCCTT	CGCTGCATCGAGATCTTCTT	93
CCL4	C-C Motif Chemokine Ligand 4	CCGTGGTATTCCAGACCAAA	ACTCCTGGACCCAGTCATCA	69
CD101	CD101	ATTGAAACTCAGGCCCACAG	CCTGGCTGTGTTCTGTAGCA	80
CD14	CD14	AAGCTCACCGTGCTTGATCT	CCTTCCAGGGTCAGGTCAT	92
CD163	CD163	CACATGTGCCAACAAAATAAGAC	CACCACCTGAGCATCTTCAA	130
CD68a*	CD68	CATGGCTGTGGAGTACAACG	TGGAGATCTCGAAGGGATGA	84
CD68b*	CD68	CTCTCATTCCCCTACGGACA	CCACAGCCATGTAGTTCAGG	85
CXCL8	C-X-C Motif Chemokine Ligand 8	CTTCGATGCCAGTGCATAAA	CAGTGGGGTCCACTCTCAAT	89
CXCL9	C-X-C Motif Chemokine Ligand 9	AGCAGTGTTGCCTTGCTTTT	ATGCAGGAACAACGTCCATT	92
CXCR2	C-X-C Motif Chemokine Receptor 2	ACAGCTGCCTCAATCCTCTC	ATGGCCATGATCTTGAGGAG	76
DDX58/RIG I	DExD/H-Box Helicase 58 or RIG-I	ACGAAAGGGGAAGGTTGTCT	ATGCCTGCAACTTTGTACCC	108
GPR84	G Protein-Coupled Receptor 84	CTGGAAGGGGACTCATCAGA	TCCTGGAGGGCTTGTCTCTA	81
HPRT1	Hypoxanthine phosphoribosyl-transferase I	ACACTGGCAAAACAATGCAA	TGCAACCTTGACCATCTTTG	71
IFNG	Interferon gamma	CCATTCAAAGGAGCATGGAT	TTCAGTTTCCCAGAGCTACCA	76
IL1A	Interleukin 1 Alpha	TGTGCTAAATAACCTGGATGAGG	GGTTCGTCTTCGTTTTGAGC	135
IL1RAP	Interleukin 1 Receptor Accessory Protein	GCATCACCTCCCCAAATCTA	GTAGCTCCTCTCCCGGTTCT	70
IRF3	Interferon Regulatory Factor 3	GCTACACCCTCTGGTTCTGC	GAGACACATGGGGACAACCT	95
IRF7	Interferon Regulatory Factor 7	GCTCCCCACACTACACCATC	TCCAACTTCACCAGGACGA	85
ISG15	ISG15 Ubiquitin-Like Modifier	AGTTCTGGCTGACTTTCGAGG	GGTGCACATAGGCTTGAGGT	80
ISG20	Interferon Stimulated Exonuclease Gene 20	AGATCCTGCAGCTCCTGAAA	TGCTCATGTTCTCCTTCAGC	84

LCN2a*	Lipocalin 2	CAGTTCCAGGGGAAGTGGTA	GAGCTCGTAGGTGGTGGTGT	99
LCN2b*	Lipocalin 2	CAGCTACAACGTCGTCTCCA	GCTGGAGACTTGGGACAAA A	80
LTF	lactotransferrin	GGAAAAGACTGCCCAGACAA	ACACTCCGTGTTGTCGTTGA	78
MX1	MX Dynamin Like GTPase 1	CCTCCACAGAACTGCCAAG	GCAGTACACGATCTGCTCCA	109
MX2	MX Dynamin Like GTPase 2	ACCAAGGGCCTGAATATGCT	ACGGGCTGTACAGGTTGTTC	100
OAS1	2'-5'-Oligoadenylate Synthetase 1	AAGAAACCCAGGCCTGTGATTC	TAGTGCCCCTTCTACCAGCT	99
OAS2	2'-5'-Oligoadenylate Synthetase 2	TTTAATTCGCCTGGTGAAGC	GCAGCTCCAAGGCATACTTC	92
OASL	2'-5'-Oligoadenylate Synthetase Like	TGCGACTGGTAAAACACTGG	CCCAGGCATAGATGGTCAG T	105
PPIA	peptidylprolyl isomerase A (cyclophilin A)	CAAGACTGAGTGGTTGGATGG	TGTCCACAGTCAGCAATGGT	138
RPL13A	Ribosomal protein L13a	ATTGTGGCCAAGCAGGTACT	AATTGCCAGAAATGTTGATG C	76
S100A12	S100 Calcium Binding Protein A12	TGAAGCAGCTGATCACCAAG	GTCTTGATTGGCATCCAGGT	101
S100A8	S100 Calcium Binding Protein A8	ATGCTGACGGATCTGGAGAG	GGCGTGGTAATTCCCTTTCT	84
S100A9	S100 Calcium Binding Protein A9	ACGAGGAGATGCACAAGACC	CACATGGGCCTGAGCTG	84
TNF	Tumor Necrosis Factor alpha	CCCCCAGAAGGAAGAGTTTC	CGGGCTTATCTGAGGTTTGA	92
VWF	Von Willebrand Factor	CACTGAAGCGCGATGAGAC	CGTGATCCTCTTCTCCCAGA	95
YWHAE	Tyrosine 3-monooxygenase/tryptophan 5-monooxygenase	GCTGCTGGTGATGATAAGAAGG	AGTTAAGGGCCAGACCCAA T	124
