# Supplementary material for: Influence of African Swine Fever Virus on Host Gene Transcription within Peripheral Blood Mononuclear Cells from Infected Pigs
Source: Viruses. 2022 Sep 29;14(10):2147. doi: 10.3390/v14102147 (PMC9610944; doi:10.3390/v14102147)
Supplement: Supplementary file 1 [file viruses-14-02147-s001.zip › viruses-1923885-Table S2.pdf]

viruses

MDPI

Table S2. Listing of the 20 genes with greatest reduction in expression within PBMCs, as determined by RNAseq, between pigs at 0 and 6 dpi. The genes are listed according to the sum rank scores based on 3 different bioconductor tools (DEseq2, EdgeR and Limma).

DE_Gene ID	DESeq2_ logFC	DESeq2 _padj	glmQLF _logFC	glmQLF _FDR	Limma _logFC	Limma _adj.P.Val	Rank1	Rank2	Rank3	Sum _Rank
NOX3	-6.74381	0.000135	-6.56849	0.01606	-4.81986	0.034462	1	1	2	4
LOC110260718	-6.21413	7.65E-06	-6.20291	0.01148	-5.38026	0.033258	2	2	1	5
LOC110257263	-5.61064	0.000103	-5.55303	0.017439	-4.71247	0.036459	3	3	4	10
LOC106504735	-5.49659	0.000551	-5.37238	0.017172	-4.2442	0.022884	4	4	6	14
CCL21	-4.86237	1.74E-11	-4.81807	0.000253	-4.76755	0.000222	8	8	3	19
LOC102163381	-5.25469	0.000196	-5.01782	0.001613	-4.23243	0.001895	6	6	7	19
HAO1	-5.33496	0.000278	-5.27811	0.015484	-3.82128	0.042666	5	5	11	21
LOC102167929	-5.04269	0.000671	-4.97942	0.021047	-4.21325	0.0349	7	7	8	22
PCD1B	-4.31016	9.46E-08	-4.28477	0.003822	-4.62769	0.007604	12	12	5	29
LOC100519521	-4.83084	2.87E-05	-4.80059	0.006259	-3.91226	0.023528	10	9	10	29
LOC106504972	-4.83507	8.37E-05	-4.77661	0.006212	-3.55474	0.024034	9	10	15	34
RGS5	-4.79051	0.000369	-4.77028	0.015871	-3.75968	0.038618	11	11	13	35
SLC34A1	-4.25146	0.000227	-4.19081	0.006682	-3.54904	0.011841	13	13	16	42
NPTXR	-3.853	1.34E-17	-3.85248	0.000105	-3.80044	0.001894	17	17	12	46
LOC110259281	-3.86791	1.76E-11	-3.89837	0.000506	-3.59405	0.004506	16	16	14	46
LHX2	-4.04741	0.000526	-3.98725	0.00687	-3.32149	0.015062	14	14	18	46
LOC110261202	-4.02963	3.82E-05	-3.98477	0.001919	-3.46823	0.004564	15	15	17	47
LOC110256271	-3.63479	2.19E-05	-3.6124	0.008617	-4.14969	0.004891	22	22	9	53
KCNK9	-3.80154	0.000943	-3.77624	0.013558	-3.04157	0.035045	18	18	24	60
ABCA6	-3.73422	3.42E-06	-3.75832	0.004383	-3.14259	0.039358	20	19	22	61
