# Supplementary material for: Influence of African Swine Fever Virus on Host Gene Transcription within Peripheral Blood Mononuclear Cells from Infected Pigs
Source: Viruses. 2022 Sep 29;14(10):2147. doi: 10.3390/v14102147 (PMC9610944; doi:10.3390/v14102147)
Supplement: Supplementary file 1 [file viruses-14-02147-s001.zip › viruses-1923885-Table S3.pdf]

viruses MDPI

Table S3. Listing of the 20 genes with the most increased expression in PBMCs, as determined by RNAseq, between pigs at 0 and 6 dpi. The genes are listed according to the sum rank scores based on 3 different bioconductor tools (DEseq2, EdgeR and Limma).

DE_Gene ID	DESeq2 _logFC	DESeq2 _padj	glmQLF _logFC	glmQLF _FDR	Limma _logFC	Limma _adj.P.Val	Rank1	Rank2	Rank3	Sum _Rank
CXCL8	7.18958	2.17E-36	7.151635	8.78E-05	6.715717	0.020313	1	1	2	4
CCL2	6.932902	6.29E-49	6.899169	4.28E-05	6.957978	0.001138	2	2	1	5
LOC100515345	6.491969	8.15E-38	6.456677	7.36E-05	6.213074	0.005303	3	3	4	10
LOC102163357	6.261555	2.25E-24	6.219334	0.000128	6.265247	0.010987	4	4	3	11
S100A8	6.142831	8.80E-44	6.125954	7.36E-05	6.105579	2.81E-05	5	5	5	15
MMP8	5.959413	3.83E-22	5.929576	0.000234	5.863027	0.019084	7	7	9	23
LOC110256045	5.950549	3.26E-31	5.912211	7.36E-05	5.870379	0.002032	8	8	8	24
HCAR2	5.964266	1.45E-32	5.931522	7.36E-05	5.760128	0.006419	6	6	12	24
CTSL	5.839068	9.78E-17	5.8001	0.00046	6.018293	0.009484	11	11	6	28
LTF	5.88709	6.27E-10	5.862242	0.001734	5.839986	0.000288	9	9	10	28
S100A9	5.853745	1.29E-42	5.838167	7.36E-05	5.79152	2.81E-05	10	10	11	31
ISG12(A)	5.45413	7.29E-14	5.455629	0.000331	5.997052	0.00013	14	14	7	35
GPR84	5.776612	3.86E-62	5.749511	1.28E-05	5.641286	0.000654	12	12	15	39
S100A12	5.735569	3.11E-41	5.720948	7.36E-05	5.705564	2.81E-05	13	13	14	40
LCN2	5.450035	5.17E-43	5.429316	6.25E-05	5.343307	4.46E-05	15	15	16	46
LOC110259710	4.990672	2.43E-08	4.953677	0.001326	5.718434	0.000818	17	17	13	47
SERPINH1	5.388786	5.84E-17	5.343537	0.000273	4.852665	0.030326	16	16	17	49
TCN1	4.875409	1.09E-11	4.84087	0.001156	4.650267	0.049502	18	18	18	54
CHIT1	4.628039	1.41E-08	4.600458	0.001595	4.570041	0.002537	19	19	19	57
UPP1	4.623115	2.59E-36	4.599011	7.36E-05	4.469	0.000134	20	20	21	61
