# Supplementary figures and images for: Influence of African Swine Fever Virus on Host Gene Transcription within Peripheral Blood Mononuclear Cells from Infected Pigs
Source: Viruses. 2022 Sep 29;14(10):2147. doi: 10.3390/v14102147 (PMC9610944; doi:10.3390/v14102147)

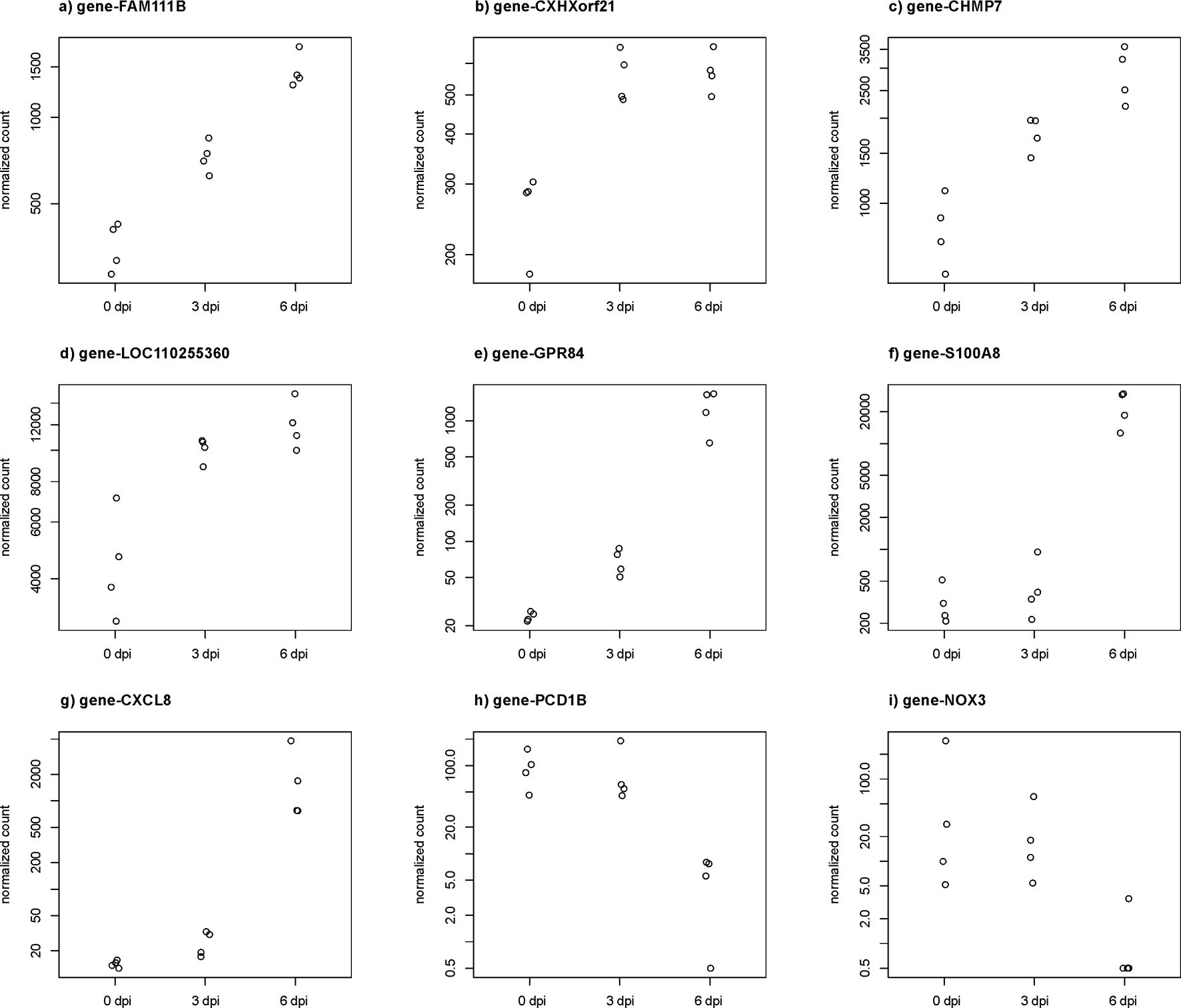

Supplement: Supplementary file 1 [file viruses-14-02147-s001.zip › viruses-1923885-Figure S1.tif]
